# Factors contributing to emotional distress in Sierra Leone: a socio-ecological analysis

**DOI:** 10.1186/s13033-021-00474-y

**Published:** 2021-06-11

**Authors:** Rebecca Horn, Stella Arakelyan, Haja Wurie, Alastair Ager

**Affiliations:** 1 Institute for Global Health and Development, Queen Margaret University, Edinburgh, UK; 2 College of Medicine and Allied Health Sciences, University of Sierra Leone, Freetown, Sierra Leone; 3 NIHR Global Health Research Unit on Health in Situations of Fragility, Queen Margaret University, Edinburgh, UK

**Keywords:** Psychosocial, Mental health, Sierra Leone, Socio-ecological, Freelisting, Qualitative

## Abstract

**Background:**

There is increasing global evidence that mental health is strongly determined by social, economic and environmental factors, and that strategic action in these areas has considerable potential for improving mental health and preventing and alleviating mental disorders. Prevention and promotion activities in mental health must address the needs prioritised by local actors. The aim of this study was to identify stressors with the potential to influence emotional wellbeing and distress within the general population of Sierra Leone, in order to contribute to an inter-sectoral public mental health approach to improving mental health within the country.

**Methodology:**

Respondents were a convenience sample of 153 respondents (60 women, 93 men) from five districts of Sierra Leone. Using freelisting methodology, respondents were asked to respond to the open question ‘What kind of problems do women/men have in your community?’. Data analysis involved consolidation of elicited problems into a single list. These were then organised thematically using an adaptation of the socio-ecological model, facilitating exploration of the interactions between problems at individual, family, community and societal levels

**Results:**

Overall, respondents located problems predominantly at community and societal levels. Although few respondents identified individual-level issues, they frequently described how problems at other levels contributed to physical health difficulties and emotional distress. Women identified significantly more problems at the family level than men, particularly related to relationships with an intimate partner. Men identified significantly more problems at the societal level than women, primarily related to lack of infrastructure. Men and women were equally focused on problems related to poverty and lack of income generating opportunities.

**Conclusion:**

Poverty and inability to earn an income underpinned many of the problems described at individual, family and community level. Actions to address livelihoods, together with improving infrastructure and addressing gender norms which are harmful to both men and women, are likely key to improving the wellbeing of the Sierra Leone population.

**Supplementary Information:**

The online version contains supplementary material available at 10.1186/s13033-021-00474-y.

## Introduction

A holistic understanding of mental health (e.g. [37]) goes beyond the absence of symptoms of mental health problems to include optimal psychological and social functioning. This indicates that mental health systems should direct resources not only towards responding to diagnosable mental disorders but on promoting and sustaining good mental health, preventing mental health problems and identifying and addressing low-level and early signs of psychological distress [29]. In recognition of this, the recent Lancet Commission on Global Mental Health and Sustainable Development [28] advocates for an expanded agenda for mental health that addresses promotion and prevention as well as treatment and rehabilitation, noting that the greatest population benefit is gained from promoting factors that facilitate good mental health and avoiding causes of ill-health. There is now a growing consensus that ‘it is time for promotion and prevention efforts to take center stage in the field of global mental health’ [32].

There is increasing evidence that mental health is strongly determined by social, economic and environmental factors [1, 21, 28], and that strategic action in these areas has considerable potential for improving mental health and in preventing and alleviating mental disorders, particularly for the underprivileged and marginalised [31]. This requires a comprehensive public mental health approach [8], with multi-layered approaches targeting various areas of need [15].

Such an approach needs to be grounded in local cultural contexts and social realities, and local perceptions of the needs that are most critical to address [29, 32]. Studies of community perceptions of the causes of psychological suffering (e.g. [10, 19, 26, 35]) typically identify a set of inter-related social and economic problems. Daily stressors have been found to have at least as much impact on mental health as extreme events such as war experiences [9, 22, 16, 18, 25, 33].

In non-conflict-affected populations, numerous studies have found that the cumulative effect of ‘daily hassles’ (defined as the lower level stressors of everyday life) is more strongly predictive of psychological distress than exposure to major life events [23]. Daily stressors commonly identified as predicting poor mental health outcomes include family violence, unemployment, perceived discrimination, food insecurity and poverty, together with broader factors such as unequal access to basic resources and opportunities to partake in occupational and recreational activities [20, 30].

### Socio-ecological framework

Tol [32] identifies the socio-ecological perspective as a key principle of prevention and promotion in mental health in low- and middle-income countries (LMICs). Widely adopted in the public health sphere [29, 30] this framework has been helpful in disentangling the reciprocal influences between the individual and the environment, providing insight into which social variables may be targeted to promote mental health and prevent mental disorders [32]. Socio-ecological framing illustrates how influences on mental health can exist at the individual level (e.g. coping styles and self-esteem), family level (e.g. parenting styles), peer, school or workplace levels (e.g. social support), community level (e.g. social capital and communal violence); and societal level (e.g. political systems; gender norms). One of the implications of a socio-ecological perspective on mental health is that a collaborative, inter-sectoral approach is required to address the inter-related factors which impact on a population’s psychological wellbeing [28, 29, 32].

### Sierra Leone

The West African country of Sierra Leone experienced a brutal civil war between 1991 and 2002, during which an estimated 70,000 people were killed and more than 2 million (more than one-third of the population) were displaced [17]. Following the war, efforts were made to rebuild systems and infrastructure within Sierra Leone, but these efforts were disrupted by the outbreak in 2014 of Ebola Virus Disease (EVD) which continued for almost 2 years, and had a devastating effect on an already fragile population. Since March 2020, Sierra Leone, along with the rest of the world, has been dealing with the effects of the COVID-19 pandemic.

People in Sierra Leone have experienced multiple adverse events in the past, combined with current struggles to maintain well-being in one of the poorest countries in the world in terms of economic development, health, and education. Sierra Leone was ranked 181 out of 189 on the Human Development Index in 2019 (United Nations Development Programme [34]).

Formal mental health service provision in Sierra Leone is limited to one psychiatric hospital in the capital city, Freetown, which receives referrals from provincial and district hospitals, NGO services and recent attempts to strengthen capacity at primary care level through the training of mental health nurses [12]. A public health approach to mental health [32] is particularly relevant to Sierra Leone because:

1. There are limitations to what a strategy focused on treatment alone can offer given the extremely limited capacity in terms of mental health professionals in the country.
2. There is now a considerable body of evidence globally around the role played by social conditions in mental health. Therefore, the burden of mental health problems in Sierra Leone is unlikely to be relieved by improved access to mental health treatments alone [21].
3. Prevention of mental health problems is more cost-effective than treatment. This is important given the very limited budget for mental healthcare in Sierra Leone.

### Aims

It is widely acknowledged that in planning mental health prevention and promotion activities in LMICs it is important to address needs which are prioritised by local actors [29, 32, 35]. This study sought to explore what adult men and women in Sierra Leone identify as the problems affecting their wellbeing to suggest potential targets of an inter-sectoral public mental health approach focusing on promoting mental wellbeing in the country.

## Method

The study is part of a larger programme of research addressing mental health and well-being in Sierra Leone (see [14]). The design of the component reported here reflects the approach of Bolton and colleagues (e.g. [2, 4, 19]) in using a freelisting methodology [39]. Freelisting is an exploratory research methodology which has been widely used to identify the priority issues affecting a population (e.g. [9]). It involves defining a broad question and briefly interviewing participants to rapidly gather information, with the frequency of the reported items used to determine salience. In-depth exploration of problems is not possible with freelisting, but it does allow for rapid exploration of local understandings which can inform future research and interventions.

### Research team

The training and supervision of the research team was carried out by a QMU researcher and coordination of logistical issues was conducted by a member of staff from the College of Medicine and Allied Health Sciences (COMAHS), University of Sierra Leone. The field researchers were all Sierra Leoneans, aged between 20 and 30 years old, and were either recent university graduates or in the final phase of their studies. They were from a number of ethnic groups, and spoke languages including Mende, Temne, Fullah and Limba as well as being fluent in Krio and English.

A team of 11 field researchers (four female, seven male) participated in a 3-day training, which consisted of sessions on research ethics and qualitative methods, plus intensive practical training in the methods to be used. This included pilot testing and revision of the methodology.

### Selection of participants

Respondents were a convenience sample selected from five districts (Bo, Kailahun, Kambia, Kono and Western Area). The locations within each district were selected in collaboration with the District Health Education Officer at the District Health Management Team to reflect diversity in terms of factors such as religion, ethnicity, socio-economic status and main form of livelihood. Initial meetings were held with local chiefs or tribal authorities to ensure access into the selected communities. Field researchers purposively sampled to ensure gender balance and a representative age range.

Freelisting interviews were conducted with a total of 153 respondents. The locations, gender and age breakdown of respondents are shown in Table 1.

**Table 1:** Respondents’ locations and ages

District	Female		Male	
	N	Age range (mean)	N	Age range (mean)
Bo	12	20–68 (38.2)	20	18–77 (39.8)
Kailahun	12	20–77 (40.3)	19	18–90 (48.6)
Kambia	12	21–66 (39.8)	18	19–80 (36.3)
Kono	12	21–82 (56.8)	18	19–82 (50.1)
Western area	12	20–60 (37.3)	18	20–78 (41.5)
**TOTAL**	**60**	**20–82 (42.5)**	**93**	**18–90 (43.2)**

### Process

Free list interviews involved asking individual respondents to provide lists of items, and brief descriptions of each item, in response to the question ‘What kind of problems do women/men have in your community?’ (female respondents were asked about problems affecting women; male respondents about problems affecting men). When the respondent had listed all the problems which came to mind, interviewers read back the list and used non-specific prompting to encourage them to think of further problems [5]. This continued until no more could be identified. Interviewers then went back through the list and asked respondents to give a brief description of each problem.

Interviewers worked in teams of two (interviewer and note-taker) to carry out the data collection [2]. Each team was allocated to a particular location, where they approached potential respondents and explained the nature and purpose of the study, including issues such as confidentiality of the information obtained and that respondents would receive no incentive or compensation for participating. Any questions were answered, and verbal consent was sought to proceed. Interviews were conducted in a location convenient to the respondent, which could be a public space (e.g. marketplace) or private home or compound, and in the respondent’s preferred language, which was primarily (91) Krio. Non-Krio interviews were mainly conducted through a translator hired by the field researchers in the local area.

Handwritten notes were taken during the interview, and reviewed afterwards by the interviewer and the note-taker to ensure that they accurately and comprehensively represented what was said by the respondent. The data were entered into an Excel spreadsheet by the lead researcher at the end of each day.

### Data analysis

Data analysis was conducted following the process described by Bolton et al. [4]. Each respondent was assigned a numeric code. All the problems identified were consolidated into a single list, with the code and gender of respondents attached to each problem they identified. At this stage, two or more respondents were recorded as having mentioned the same problem if they referred to it using the same language. The resulting list was then reviewed by the research team to identify problems that were similar in meaning but had different wording. Where this occurred, the most clearly worded version (based on a consensus among the research team) was retained to represent all the versions. The respondent code numbers for the deleted response were then added to those of the retained version so that all the respondents who reported the problem were accounted for.

The reported problems were collapsed into themes and organised into ecological levels using an adaptation of the socio-ecological framework [6]. The first author developed criteria to determine how problems would be assigned to each level of the socio-ecological framework. These criteria were reviewed by the second author, and were revised through discussion between the two authors. The problems were then assigned to each level of the framework by the first author, then reviewed by the second author. Discrepancies were discussed and a solution arrived at through consensus. The use of the socio-ecological model facilitated (a) an exploration of the interactions between the problems at individual, family, community and societal levels, and (b) a comparison of frequency distributions for identified problems at each ecological level by gender. For the latter, the Fisher’s exact test of independence was used given the sample size and that expected values in some of the cells of the contingency table were below 5 [11].

## Results

**Fig. 1:**
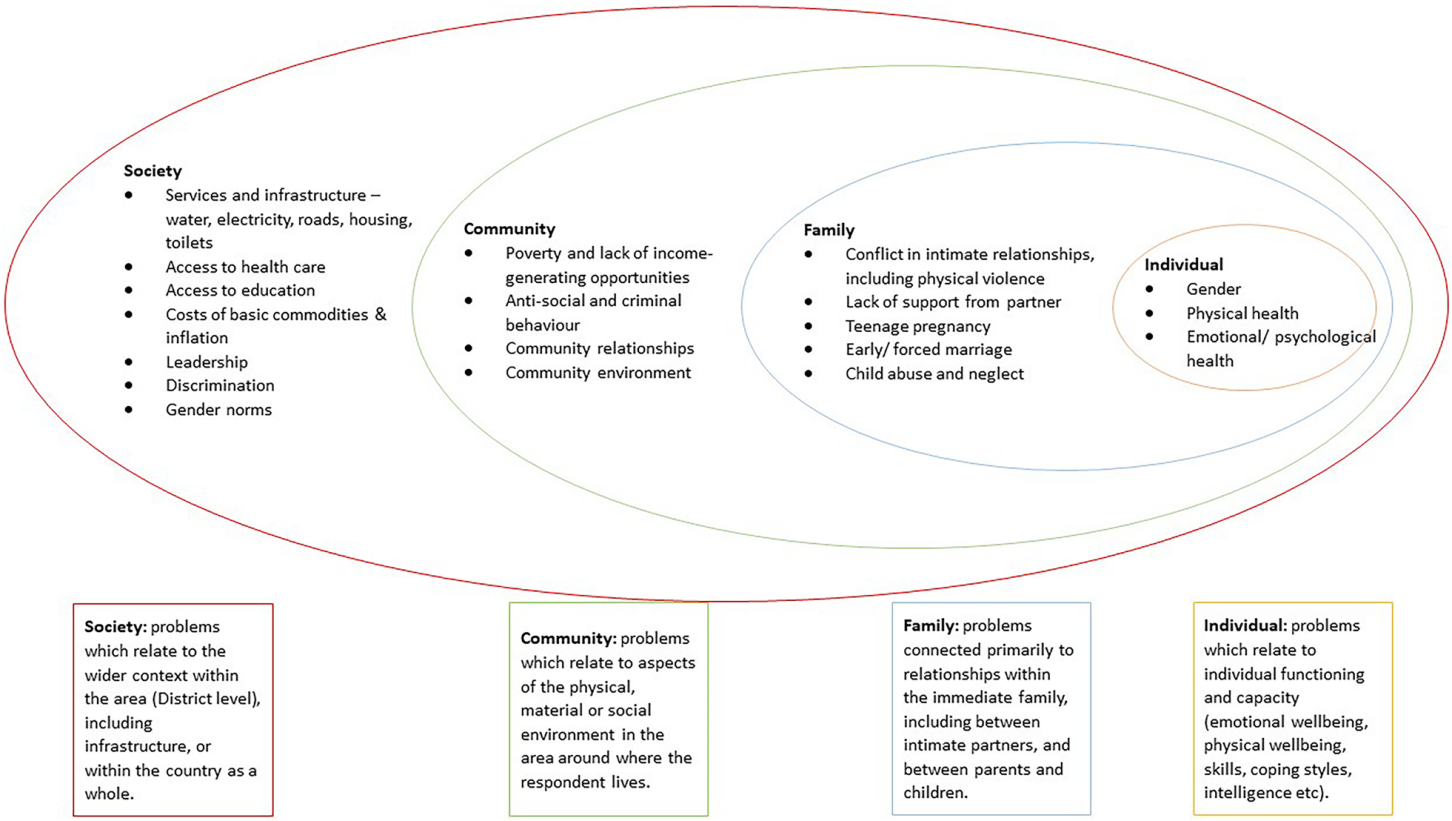
Socio-ecological model illustrating range of problems identified

A total number of 1124 problems were identified. Individual respondents identified between 1 and 12 problems, with the mean number of problems identified being 7.4. Problems identified by fewer than three respondents were excluded. The remaining problems were collapsed into 43 themes and organised into the socio-ecological levels shown in Fig. 1.

**Fig. 2:**
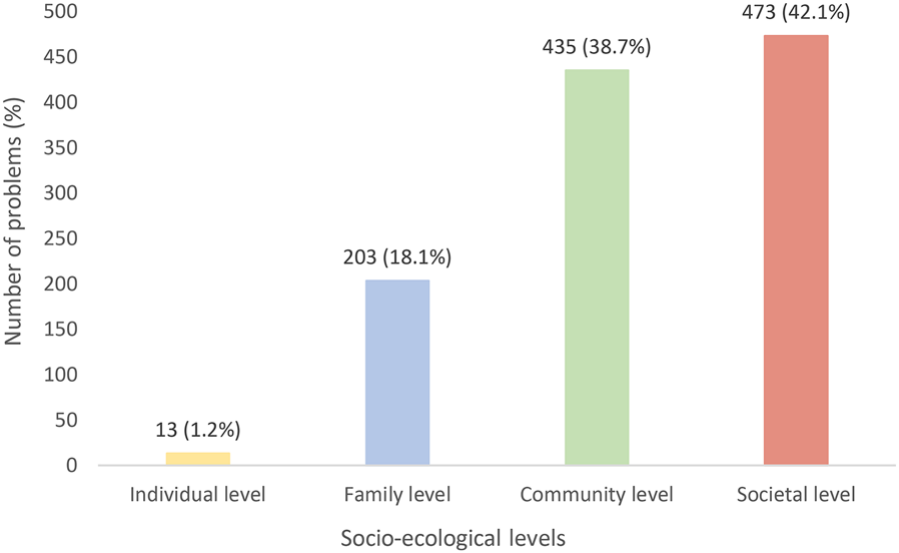
Number of problems identified at different levels

Overall, there was a clear trend for more problems to be located at more distal socio-ecological levels (see Fig. 2). Table 2 shows the distribution of problems identified at each level disaggregated by gender. Although the overall number of individual problems identified was small, men were marginally more likely to identify issues at this level than women (p = 0.05). Otherwise, women were significantly more likely to locate problems at the level of the family (p < 0.001) than men, while men located more problems at the societal level than women (p = 0.01). There were no gender differences observed in attributing problems at community level (Table 2).

**Table 2:** Frequency distributions of problems within socio-ecological levels by gender

Socio-ecological levels		Men (n = 93)	Women (n = 60)	Fisher’s exact test
Level	Number of problems	Frequency (%)	Frequency (%)	Exact Sig. (2-sided)
Individual	0	82 (88.2%)	59 (98.3%)	P = .051
	1	10 (10.8%)	1 (1.7%)	
	2	1 (1.1%)	0 (0%)	
Family	0	81 (87.1%)	3 (5.0%)	P < .001
	1	11 (11.8%)	13 (21.7%)	
	2	1 (1.1%)	12 (20.0%)	
	3	0 (0%)	10 (16.7%)	
	4	0 (0%)	12 (20%)	
	5	0 (0%)	4 (6.7%)	
	6	0 (0%)	2 (3.3%)	
Community	0	3 (3.2%)	3 (5.0%)	P = .775
	1	17 (18.3%)	13 (21.7%)	
	2	25 (26.9%)	12 (20.0%)	
	3	21 (22.6%)	10 (16.7%)	
	4	11 (11.8%)	12 (20.0%)	
	5	11 (11.8%)	6 (10.0%)	
	6	3 (3.2%)	3 (5.0%)	
	7	2 (2.2%)	1 (1.7%)	
Society	0	6 (6.5%)	9 (15.0%)	P = .014
	1	7 (7.5%)	12 (20.0%)	
	2	16 (17.2%)	13 (21.7%)	
	3	15 (16.1%)	14 (23.3%)	
	4	21 (22.6%)	6 (10.0%)	
	5	14 (15.1%)	3 (5.0%)	
	6	8 (8.6%)	2 (3.3%)	
	7	4 (4.3%)	1 (1.7%)	
	8	2 (2.2%)	0 (0%)	

### Individual factors

As noted, few problems were identified at the individual level by either women or men. Those mentioned were either physical health problems (identified ten times by men) or emotional problems (identified once by women and twice by men), such as the pain of losing a loved one (identified by the female respondent) and distress caused by poverty and the inability of taking care of basic needs (identified by the two male respondents).

### Family factors

The majority of problems at family level related to conflict within intimate relationships, including physical violence, with 17% of all the problems said to affect women falling into this category. These issues were highlighted by women across all districts, and across the whole age range of female respondents.

All four men who said that relationship problems affected men in their community, and three women, referred to the effects of lack of income-generating opportunities and poverty on relationships, when men are unable to provide for the needs of their family in the way expected.

‘When men cannot take care of their family this leads to unrest in the home and disrespect from their wife’ (38-year old man, Kambia district).

‘When women know they are the breadwinner of the home they no longer respect their husband and husband cannot take it so they fight’ (82-year old woman, Kono district).

Most women who identified relationship conflict as a problem in their community related it to men having more than one wife or being unfaithful.

‘Marital problems are too much, our men cheat, they disrespect us and they don't treat us as equals’ (65-year old woman, Kono district).

Physical violence within intimate relationships was identified by 28 women and no men as a problem. Some of the women said that the violence occurred due to conflict over financial issues or infidelity, as described above, but the majority attributed the violence to prevailing gender norms in the society (at the ‘societal’ level of the socio-economic model).

‘Men like bullying women, they think we don't have freedom, they feel like they are the boss and have power over us’ (35-year old woman, Kambia).

‘Women are beaten and maltreated at home and all they will advise is to stay and obey your husband if you want to be married’ (23-year old woman, Kono district).

Some female respondents specifically related women’s mistreatment by men to economic issues, stating that because women were unable or unwilling to earn their own money they were more vulnerable.

Women who talked about conflict in relationships and intimate partner violence described the effects on their emotional and physical wellbeing (individual level).

‘Violence against women makes women to be so insecure, low self-esteem and ashamed. Some men beat their women to death’ (20-year old woman, Bo district).

Women also referred to problems due to the lack of support from a male partner (over 5% of all problems affecting women), including men not taking responsibility for their families, women being abandoned by their partners, being widowed or unmarried. Respondents described how women in this situation were left to shoulder the burden of caring for the family alone.

‘The men just abandon us here. We the women labour for ourselves to feed and clothe our children’ (52-year old woman, Kailahun).

‘Our children depend on us and the men are not helping us in taking care of them’ (35-year old woman, Kono).

A woman being unmarried was said to have consequences on women’s wellbeing both economically and socially. The stress of having to provide financially for one’s children without any support was mentioned by several women, and those who were without partners through abandonment, divorce or never having married were said to be particularly socially marginalised.

‘Women who have lost their husband are lonely. They are most times bullied and marginalised by society’ (54-year old woman, Bo district).

Problems experienced by younger women, particularly teenage pregnancy and early marriage were mentioned primarily by female respondents, and were identified more often in rural areas than in the two more urbanised districts (Western Area and Bo).

Young women and girls were said to be sometimes forced into early marriages primarily because their families could not afford to keep them. Lack of income was also related by some respondents to teenage pregnancy, in that children were unwilling to listen to or abide by the rules of parents who were unable to provide for their needs.

‘Majority of our children and grandchildren get pregnant in school because they don't listen to us because we cannot take care of them’ (62-year old woman, Kono district).

Some women and a small number of men expressed concerns about the relationship between children and their families, with some describing children being abused, neglected and/ or exploited, and others concerned that children and youth lacked respect for their parents and families. Six of the eight occasions where child mistreatment was identified as a problem were from respondents in Western Area, which is more urban than the other districts.

‘No care for children and women give birth to more children and leave them in the street’ (27-year old woman, Western Rural).

As noted above, relationships between children and their families were said to be influenced by poverty, with children not respecting parents who were unable to provide for their needs. However, a few respondents also said that parents who had financial problems mistreated their children by sending them out to work (or forcing them into early marriages, as described above) or not making efforts to support them.

### Community factors

Poverty and the inability to meet basic needs was referred to very frequently by men (it accounted for more than one-fifth of all problems said to affect men) and a considerable proportion of women. The numbers referring to this issue was roughly equivalent across all five locations.

‘The children depend on us and we don’t have any access to money’ (35-year old woman, Kono district).

The most commonly-cited reason for this, especially outside Western area, was a lack of jobs even for those who had skills and qualifications. In Kono district, employment was affected by the closure of the mining operations during the Ebola outbreak, many of which did not re-open afterwards. In other districts, participants said their area was under-developed (e.g. poor road network) which restricted businesses from operating in that location.

‘Men find it really difficult to get a job after graduating from college or after learning a skilled job’ (23-year old man, Kambia district).

‘No company or factory operate here to provide job for people and most of the NGOs have also left the city’ (55-year old man, Kailahun district).

A related challenge in earning a basic income was that even if somebody did manage to start a business, or to sell products they had grown or made, business was slow because the general level of poverty meant that there were few customers or markets.

‘The materials are expensive and it takes long for people to buy our furnitures’ (19-year old man, Kono district).

In many cases, though, people were unable to start businesses because they did not have the capital to do so, did not have the skills and/ or were unable to produce crops for sale because of challenges they faced in farming (e.g. poor soil, lack of tools or seeds). Some noted the lack of support from government or non-governmental agencies which may have helped them to overcome some of these barriers.

‘There is no money, no help from government. For us the women, women’s organisations are not focusing here’ (56-year old woman, Kambia district).

The solution chosen by many was to take up some form of casual work; this was often motorbike riding (motorbike taxis or *okadas*) for the young men, or in some locations farming, sand mining, stone mining or informal diamond mining. Women also undertook casual work where possible. Only female respondents (14 women, mainly in Kailahun and Western Area) identified commercial sex work as a problem affecting their communities, and this was almost always said to be related to a lack of alternative sources of income for young women.

‘Women want money so they sleep with all men, even married men and destroy the relationships around’ (27-year old woman, Western Rural).

In addition to lack of money to buy food and other essentials, in some areas there were also inadequate food supplies even for those who did have money. This was mentioned in all areas, but especially Kailahun and Kambia districts. In some cases this was because farming was poor in that area, and others referred to food shortages at particular times of year (the dry season).

As well as being unable to eat regularly or well, the lack of income-generating opportunities was said to have consequences for the social wellbeing of individuals and communities. The impact of lack of income on family relationships was discussed earlier, and parents were in some cases unable to meet the costs of sending their children to school. Idleness, especially of youth, was said to lead to emotional distress (frustration) and in some cases criminal behaviour and lawlessness.

‘Because there are no jobs, these young boys are idle and it leads to many problems’ (51-year old male, Bo district).

Men were highly affected, and women to a lesser extent, by a cluster of problems related to anti-social and criminal behaviour. This included general insecurity and theft, as well as violence within the community. There was some reference to *kliks* or gangs, which were said to contribute to general lawlessness in communities and well as conflict between different *kliks*.

‘The young boys join *kliks* and fight with each other in rival *kliks*’ (51-year old man, Bo district).

These *kliks* were sometimes held responsible for thefts within the community, but such behaviour was also said to be due to a lack of job or business opportunities for young men, as described above. High crime rates were said to cause stress, anxiety and feelings of insecurity for those affected or at risk.

‘The youth are in the habit of doing that. They sit on the bikes taking peoples bags, purses, breaking into houses and shops’ (45-year old woman, Western Area).

In addition to criminal behaviour, respondents expressed concern about a general feeling of lawlessness in their communities, and violence amongst the youths themselves. This was sometimes related to conflict between the *kliks*, or in relation to football or politics. Fighting between young girls and between families was also referred to. Violence in general within communities was a concern for these respondents.

‘There is too much violence in this community, especially among the youth’ (28-year old man, Bo district).

Sexual violence against women was referred to by nine women and two men, who were located across all five districts. This included exploitation of women and girls by men in more powerful positions.

‘Women are harassed on a daily basis. Men want sex for every little favour women ask from them. Women are not happy about this’ (54-year old woman, Bo district).

‘Not good enough caring from parents so they leave their children to go out on the street wearing attractive things, so because of that some men cannot control themselves so they rape them’ (25-year old woman, Western Rural).

Substance use was often mentioned alongside these issues. For some, there was a general concern about the level of substance use, particularly alcohol, cigarette smoking, marijuana smoking and drugs (tramadol), which was seen as a general sign of disrespect for the community, as well as contributing to other forms of disruptive behaviour. For others, they expressed particular concern about the levels of alcohol or tramadol being taken and the relationship with violence.

‘Alcohol abuse is very common here, and this causes the men to be involved in violent acts’ (28-year old man, Bo district).

‘Boy and girl now involved in drinking and smoking around, which is not good and after drinking they involve in bad habit’ (62-year old woman, Kono district).

Women, much more than men, identified problems relating to poor relationships within the community. These related primarily to women discussing the behaviour, circumstances and appearance of other women, including telling lies about other women, which created tensions and conflict. Some saw this behaviour as being related to idleness, when women did not have jobs or businesses, whilst others related it to jealousy.

‘Women are engaged in gossip, they sit talking about people's business, family or if something bad happen to someone, if there is a little argument they spill everything out that they were gossiping about’ (38-year old woman, Kailahun district).

‘Because there is nothing to do, they tend to become idle, no job, talk around, which causes quarrelling and argument’ (27-year old woman, Western Rural).

### Societal factors

More than half the problems identified by male respondents were at this level of the socio-ecological model, compared to just over one-quarter of the problems identified by women. The majority of the challenges described at this level related to a lack of services and infrastructure.

Problems related to access to water were identified across all districts. In these communities there was either no pipe-borne water taps (so people had to use river water or walk long distances) or insufficient water for the population. The situation was especially difficult during the dry season, when water sources might dry up. This resulted in people drinking unclean water, going long distances to fetch water and/ or paying for water.

‘Most of the pumps in this community need repair. We even pay for drinking water, because we only have one pump now’ (25-year old woman, Kambia district).

Respondents from all five areas of the country identified a lack of toilets as a problem affecting their community. Households did not tend to have their own toilets, they shared and there was a lack of public toilets. Many respondents said that the toilets in their community were broken or full because of the number of people using them. The consequence of this was that people went to the bush to defaecate, contributing to sickness within the surrounding communities.

‘We have only one government toilet in the community, which is not enough for us. We most of the time use the bushes’ (37-year old man, Kambia district).

A lack of electricity was an issue identified across all districts, with some respondents concerned about the effect of this on business and the development of their community. Problems related to housing were also identified across all areas, with a higher proportion from Bo district and Western Area (the more urban districts). Some highlighted the high cost of rent, connecting to the lack of income-generating opportunities but the issue mentioned most often was the poor condition of housing which led to health problems.

‘The houses in this community are not properly built, which leads to mosquitos and cockroaches gaining easy access inside our homes’ (28-year old man, Kono district).

Respondents across all five areas identified a lack of community facilities as being a problem. Respondents particularly in Bo, but also in Kailahun and Kono, said there was a lack of a space for community members to gather for meetings and to host visitors, including a place for youth to gather. The other main community facility said to be lacking was a market. This was highlighted by as many women as men, in contrast to other issues at the societal level.

‘our market is not built, we try to build it with sticks but when it’s rainy season it's really not easy’ (24-year old woman, Kailahun district).

A lack of access to good healthcare was of concern to men and women across all five areas. For some cost was said to be the main barrier to accessing healthcare, even for services which should be free. However, more commonly mentioned (especially in Bo and Kailahun) was the lack of health facilities within the local area where respondents lived, meaning that they had to travel some distance to access the care they needed. This had implications in terms of the cost of transport, and the time taken to reach a healthcare facility in case of emergencies.

‘This is a big community but we don't have clinics or health facilities here, which is a serious problem’ (27-year old man, Bo district).

For some of those who did have access to healthcare facilities, they felt that it was not adequate in terms of the staff availability and competencies, and/or the medication and equipment available.

A lack of access to educational services was identified as a problem affecting men in all five areas, and, to a lesser extent, women. In a number of cases, the barrier to accessing education was cost, despite the fact that education should be freely provided, as with healthcare. In some areas, there was a lack of local government schools meaning that those that were available were very overcrowded, or that children had to walk a considerable distance to attend school.

‘We lack schools because we only have one structure and it can't accommodate all our children’ (24-year old woman, Kailahun district).

Men also identified a lack of vocational training or adult education services as a problem, perhaps related to their concern at the lack of income-generating opportunities.

Poor road networks were identified as a problem affecting men in all five areas, and, to a lesser extent, women.

‘The roads are very bad, especially in the raining season. Most cars or public transport don’t come here during that time’ (55-year old woman, Kambia district).

The poor road networks contributed to poverty since the cost of food which had to be transported into the area was higher, and public transport costs were also higher. In some areas, there were limited public transport vehicles, which was attributed in part to the poor state of the roads. Poorly maintained roads were said to contribute to health problems, as the risk of accidents increased and the dust from the roads caused respiratory problems.

High costs in general, and of food in particular, were also identified as a problem at this level. This was evident in all areas, but particularly in Kailahun, Kambia and Kono. Where respondents identified a reason for this problem, it was most commonly inflation.

The cost of non-food commodities, including fuel and building materials, was said to be increasing and this limited the amount of food families were able to buy with their limited income. The increased cost of fuel also had an impact on the availability and cost of public transport. The cost of farm equipment (tools and fertiliser) was said to hinder crop production, which again had an impact on the availability and cost of food.

‘The food stuffs are not plenty in the market, so they are expensive’ (33-year old man, Kailahun district).

## Discussion

The problems which men and women in Sierra Leone identified as affecting their communities were predominantly situated at the family and community and societal levels of their worlds, and were highly interrelated and often fuelled by harmful societal and gender norms. Although few respondents identified individual-level factors specifically, they often described how problems at other levels contributed to physical health and wellbeing problems and emotional distress.

The focus on problems at the family, community and societal levels is in line with findings from a study conducted involving young people in Sierra Leone [9] and from other contexts [19, 35]. Typically, people are more concerned with social and economic problems than with explicitly psychosocial or mental health issues, but recognise the inter-related nature of these issues.

The gendered nature of the responses was apparent, with women reporting to a much greater extent than men that they were affected by problems at the family level. Whilst both men and women reported being affected by problems at the community and societal levels, a greater proportion of the problems reported by men related to poverty or lack of income-generating opportunities and poor infrastructure. There is a clear link with gender norms at societal level, with expectations of both men and women impacting on their wellbeing. For men, the pressure to provide for their families in the absence of income-generating opportunities was an important problem, and contributed in some cases to tensions and conflict within the family, so affecting the wellbeing of all household members. In other contexts, employment has been found to be an important protective factor against mental disorders, especially for men [21]. Some female respondents explicitly referred to gender norms as a problem for women, and others did so implicitly when they explained how dependence on men, both financially and socially, caused problems for those who were abandoned by their partners, widowed or unmarried. Households headed by women caring for children in the absence of a male partner were reported as being marginalised within their communities,a situation likely to also have negative impacts on the emotional and physical wellbeing of their children. Research on intimate partner violence in Sierra Leone [13] links women’s financial dependence on men to their inability to leave abusive relationships. Patel et al. [28] refer to studies in various settings which have shown that ‘gender disempowerment interacts with other adversities such as poverty, gender-based violence, sexual harassment and food insecurity to increase the prevalence of common mental disorders in women’ (p. 14).

The position of women in Sierra Leone has been a cause for concern for some time, leading to a number of initiatives designed to uphold women’s rights. Sexual violence against women and girls is widespread in the country. The high levels of illiteracy, economic insecurity and poverty amongst Sierra Leonean women collectively has disempowered women, deterring them from understanding and upholding many of their rights. Recent political initiatives to address this include the “Hands off our Girls” campaign, launched by the current First Lady of Sierra Leone together with First Ladies of other African countries and local girls’ rights champions. In February 2019, “a State of Public Emergency over rape and sexual violence” was declared but was revoked by Parliament in June 2019. In September 2019, the Parliament passed the Sexual Offences Amendment Act, which enables more stringent penalties for sexual offence cases to be imposed by the courts. The Gender Equality and Women’s Empowerment policy, launched by the Minister of Gender and Children's Affairs in December 2020, seeks to address gender inequalities, minimise poverty levels and incidences of social injustices, and enhance public and private investment to create a society in which all citizens have equal access to basic services and enjoy the same rights and opportunities in enabling environments. The embedded nature of harmful gendered norms creates challenges in achieving the aims of these initiatives; a multi-sectoral approach including both bottom-up and top-down elements is necessary for their success.

Infrastructure and the physical environment are often overlooked as factors that impact on psychological wellbeing, but our findings suggest that, especially for men, they are perceived to be important. This is in line with Allen et al.’s [1] review of the social determinants of mental health, which concludes that access to basic amenities such as water, sanitation and waste management improvements, interventions such as energy infrastructure upgrades, new transport infrastructure, mitigation of environmental hazards, and improved housing can improve mental health and functioning. Similar findings have been obtained in very different settings. For example, Panter-Brick and Eggerman [27] conclude that in the Afghan context, a culturally relevant mental health intervention would focus on providing structural, social and economic resources to families who struggle with everyday stressors.

Our socio-ecological framing highlights the inter-related nature of problems. In terms of strengthening mental health systems, this indicates that identifying and addressing priority problems at one level of the model is likely to have positive consequences not only on other factors within that level, but also across levels. For example, strengthening income-generating opportunities for both men and women is likely to have positive consequences within the community level as youths involved in anti-social behaviours will be engaged in constructive activity and able to earn an income, but also in terms of family relationships, including the wellbeing and development of children, and on individual physical and psychological health. Miller and Rasmussen [23] suggest that those daily stressors that are particularly salient and can be affected through targeted interventions should be addressed as a priority, since this will reduce the proportion of the population which will require specialised clinical services [3]. By promoting good psychological wellbeing through improving the quality of the social and material environment, it will be easier to identify those whose distress remains high and who require more focused interventions (whether from mental health specialists or others).

Although this study did not aim to investigate the relationship between individual experience of problems and levels of distress, there is considerable evidence from other contexts that this relationship does exist (e.g. [1, 21, 22]). In a country such as Sierra Leone, where the capacity for specialised mental health supports is extremely limited, there is a strong argument for focusing resources on improving the economic situation and infrastructure, and strengthening relationships within families and communities, in order to promote good mental health and prevent the development of the more severe forms of distress. It is also important that measures should be put in place to challenge some of these harmful gender and societal norms as a gradual but steady process, for example through the work of Community Health Workers who are active throughout the country.

This would also reduce the low-level chronic distress that often results from the type of ongoing non-traumatic daily stressors [23] described by respondents in this study, and which has been found to be associated with more total disability at a population level than diagnostically defined mental disorders [7]. Identifying the key problems affecting people in Sierra Leone, and ways in which inter-sectoral initiatives within the country could address these issues, has the potential to prevent more severe psychological problems and contribute to productivity within the country. This requires professionals in different sectors—including governmental agencies, nongovernmental organisations, private sector organisations, social institutions, community and voluntary groups—to coordinate their efforts and integrate mental health into a broad range of related policy areas [30]. This is in line with the World Health Organization (WHO) ‘intersectoral action for health’ that calls for collaboration by highlighting the importance of a relationship between different health sectors and other sectors for improving health outcomes in a more effective, efficient and sustainable way [36]. The involvement of community actors in this process is crucial. Tol [32] notes that effective prevention and promotion interventions build on the resources that exist within communities, prevention and promotion ‘needs national policies, but local actions’ [1].

Mental health practitioners in some parts of the world (Latin America, Palestine) have long argued for political advocacy to achieve structural changes linked to better mental health, and Miller and Rasmussen [24] suggest that a more central role for advocacy is essential to achieve lasting improvements in mental health and psychosocial wellbeing.

In Sierra Leone, the findings of this study contribute to advocacy in two significant areas: addressing gender norms and inequities which have a negative impact on wellbeing; and establishing a coordinated, multi-sectoral effort to promote good mental health and psychosocial wellbeing.

As noted previously, there are concerns regarding the position of women in Sierra Leone. However, the findings of this study indicate that the current entrenched gender norms create problems not only for women but also for men, who are often unable to fulfil societal expectations, particularly in relation to providing for the material needs of their families. This contributes to tensions in the home and family conflict and violence, which in turn leads to emotional and physical health problems. The inter-related effects of gender expectations and norms in Sierra Leone have a clear connection to mental health issues, and any public health strategy must take these into account.

In order to address this and other issues contributing to the mental health and psychosocial wellbeing of the Sierra Leonean population, a multi-sectoral, multi-level coordinated effort is essential. The findings of this study indicate that actors with responsibility for transport networks, water and sanitation, education, social welfare and gender issues all have a role to play in addressing mental health issues, in addition to that of the health sector. Collaborative, multi-sectoral policy implementation in Sierra Leone would promote the effectiveness of policies relating to cross-cutting issues such as mental health, psychosocial wellbeing and gender issues. Historically, there has been a disconnect between implementation at national, regional and local levels, leading to fragmented efforts with little sustainable impact. One of the factors contributing to this is short-term support from partners on specific projects, implemented at local level and not embedded in systems which will continue after projects are completed. During the Ebola outbreak in Sierra Leone, lessons were learned about the importance of Ministries and other bodies working collaboratively towards a common goal, and similar approaches will be required in order to strengthen the mental health and psychosocial wellbeing of the population.

### Limitations

The freelisting method has limitations, primarily relating to the fact that it does not permit in-depth exploration. Whilst the methodology had great utility in an exploratory study designed to learn about local perspectives in a relatively short period of time, it did not enable systematic study of the relationships between the different problems identified (or the levels of the socio-ecological model).

Tol [32] and others (e.g. Patel et al. [28]) have emphasised the importance of taking a developmental perspective on prevention and promotion in mental health in LMICs. We did not address age-related diversity within the data. Further research in this area should explore not only age differences within the adult population of Sierra Leone, but also problems experienced by young people and children, building on the work conducted by Efevbera and Betancourt in 2008 and 2010 [9].

The small number of respondents in each location make it difficult to draw conclusions about within-country differences in priority issues, and these should also be explored more fully in future studies.

## Conclusions

Poverty and inability to earn an income underpinned many of the problems described at individual, family and community level. Actions to address this issue, together with improving infrastructure and addressing gender norms which are harmful to both men and women, will contribute substantively to the wellbeing of the Sierra Leone population, and in turn contribute to the development of the country.

The next phase of our work will investigate the relationship between social determinants and levels of distress in Sierra Leone. This will enable the identification of the aspects of people’s lives which (individually and in combination) contribute most significantly to levels of distress, and so should be prioritised by bodies aiming to strengthen mental health systems in Sierra Leone.

## Supplementary Information

**Additional file 1:** **Table S1**. Problems identified at the family level. **Table S2**. Problems identified at the community level. **Table S3**. Problems identified at the societal level.

## Data Availability

The datasets generated and analysed during the current study are available from the corresponding author on reasonable request.
