# Supplementary material for: Factors contributing to emotional distress in Sierra Leone: a socio-ecological analysis
Source: Int J Ment Health Syst. 2021 Jun 11;15:58. doi: 10.1186/s13033-021-00474-y (PMC8193165; doi:10.1186/s13033-021-00474-y)
Supplement: Supplementary file 1 — Additional file 1: Table S1. Problems identified at the family level. Table S2. Problems identified at the community level. Table S3. Problems identified at the societal level. [file 13033_2021_474_MOESM1_ESM.docx]

**Additional file 1**

Table S1. Problems identified at the family level

| **FAMILY LEVEL** | **WOMEN**  **N problems (%)** | | **MEN**  **N problems (%)** | |
| --- | --- | --- | --- | --- |
| Relationship/ marital problems | 57 (11.4) | 4 (0.6) | |  |
| Teenage pregnancy | 38 (7.6) | 4 (0.6) | |  |
| Domestic violence/ gender violence | 28 (5.6) | 0 | |  |
| Early marriage | 16 (3.2) | 1 (0.2) | |  |
| Widows/ being unmarried/ single mothers | 15 (3.0) | 0 | |  |
| Women abandoned by men/ not taking responsibility | 11 (2.2) | 1 (0.2) | |  |
| Women depend on men | 8 (1.6) | 0 | |  |
| Child abuse/ child labour/ child neglect | 7 (1.4) | 1 (0.2) | |  |
| Children/ youth lack respect for parents and family | 5 (1.0) | 2 (0.3) | |  |

Table S2. Problems identified at the community level

| **COMMUNITY LEVEL** | **WOMEN**  **N problems (%)** | **MEN**  **N problems (%)** |
| --- | --- | --- |
| Lack of income generating opportunities | 54 (10.7) | 133 (21.2) |
| Alcohol/ drugs/ smoking | 15 (3.0) | 25 (4.0) |
| Insecurity/ stealing/ robbery | 11 (2.2) | 24 (3.8) |
| Gossip/ quarrelling/ lies/ back-biting | 32 (6.4) | 1 (0.2) |
| Inadequate food available | 15 (3.0) | 16 (2.6) |
| Violence/ fighting | 8 (1.6) | 12 (1.9) |
| Poor environmental cleanliness/ care | 1 (0.2) | 14 (2.2) |
| Commercial sex work | 14 (2.8) | 0 |
| Lawlessness | 2 (0.4) | 9 (1.4) |
| Rape/ sexual assault | 9 (1.8) | 2 (0.3) |
| Elders not respected | 1 (0.2) | 6 (1.0) |
| Secret society issues | 5 (1.0) | 2 (0.3) |
| School drop out | 0 | 6 (1.0) |
| *Kliks* (gangs) | 2 (0.4) | 3 (0.5) |
| Poor soil for farming | 0 | 3 (0.5) |
| Overcrowding | 2 (0.4) | 1 (0.2) |
| Gambling | 0 | 3 (0.5) |

Table S3. Problems identified at the societal level

| **SOCIETAL/ INFRASTRUCTURE LEVEL** | **WOMEN**  **N problems (%)** | | **MEN**  **N problems (%)** | |
| --- | --- | --- | --- | --- |
| Lack of water | 27 (5.4) | 66 (10.5) | |  |
| Lack of access to good health care | 24 (4.8) | 42 (6.7) | |  |
| Lack of community facilities | 20 (4.0) | 34 (5.4) | |  |
| Lack of access to education | 11 (2.2) | 30 (4.8) | |  |
| Poor road network | 5 (1.0) | 33 (5.3) | |  |
| Poor toilet facility | 15 (3.0) | 23 (3.7) | |  |
| No electricity | 4 (0.8) | 33 (5.3) | |  |
| High costs | 7 (1.4) | 18 (2.9) | |  |
| Housing problem | 9 (1.8) | 16 (2.6) | |  |
| Lack of access to transportation | 5 (1.0) | 11 (1.8) | |  |
| Poor leadership | 1 (0.2) | 10 (1.6) | |  |
| High cost of food | 2 (0.4) | 8 (1.3) | |  |
| Gender norms | 8 (1.6) | 0 | |  |
| Discrimination/ injustice | 1 (0.2) | 6 (1.0) | |  |
